# Late-systolic to early-diastolic hemodynamic force analysis in ischemic heart disease using vector flow mapping and invasive pressure measurement

**DOI:** 10.1007/s00380-026-02664-5

**Published:** 2026-02-16

**Authors:** Takushi Sugiyama, Naoyuki Otani, Haruka Noma, Harunori Takahashi, Takanori Yasu, Yasuhiro Maejima

**Affiliations:** 1 Department of Cardiovascular Medicine and Nephrology, Dokkyo Medical University Nikko Medical Center, Nikko, Tochigi Japan; 2 Department of Cardiology, Dokkyo Medical University Nikko Medical Center, Nikko, Tochigi Japan; 3 Department of Cardiology, NHO Yonago Medical Center, Yonago, Japan; 4 Department of Clinical Laboratory, Dokkyo Medical University Nikko Medical Center, Nikko, Tochigi Japan; 5 Department of Rehabilitation, Dokkyo Medical University Nikko Medical Center, Nikko, Tochigi Japan

**Keywords:** Ischemic heart disease, Left ventricular aneurysm, Vector flow mapping, Hemodynamic forces, Apical suction

## Abstract

Hemodynamic force (HDF) analysis using vector flow mapping (VFM) enables noninvasive assessment of intraventricular pressure differences (IVPDs). In this study, we evaluated the apical suction from late systole to early diastole in patients with ischemic heart disease (IHD) with or without left ventricular aneurysm (LVA), validated against simultaneous left atrial–LV pressure measurements. A total of 37 participants were evaluated: 11 healthy controls, 11 patients with IHD without LVA, and 15 with LVA. VFM-derived IVPDs were quantified as IVPD recoil (late systole) or IVPD RF (early diastole). Patients with LVA showed markedly reduced IVPD recoil and the combined parameter (IVPD_VFM_ recoil + RF) compared with those with IHD, indicating impaired apical suction. IVPD RF and the combined parameter (IVPD_VFM_ recoil + RF) correlated with transmitral E-wave (*r* = 0.54, *P* < 0.01 and *r* = 0.45, *P* = 0.03); the combined parameter (IVPD_VFM_ recoil + RF) showed significant correlation with catheter-derived simultaneous pressure differences (*r* = 0.40, *P* < 0.05). These findings demonstrate that VFM identifies loss of apical suction in LVA and reflects impaired LA–LV driving force. VFM offers a practical noninvasive tool for evaluating diastolic suction beyond Doppler indices and may improve physiological assessment in post-infarction LV remodeling.

**Supplementary Information:**

The online version contains supplementary material available at https://doi.org/10.1007/s00380-026-02664-5.

## Introduction

The assessment of cardiac physiology has traditionally relied on pressure-volume analyses, whereas hemodynamic force (HDF) remains a less established clinical indicator. Cardiac blood motion reflects ventricular function [1–4]; however, practical imaging methods to quantify this flow are limited. HDF analysis estimates intraventricular pressure differences (IVPDs), which drive blood flow and are determined by coordinated interactions among the myocardium, valves, and great vessels [5]. Although IVPDs have been invasively measured in animal models [6], noninvasive approaches, such as time-resolved magnetic resonance imaging [7] and echocardiographic particle image velocimetry [8], have recently emerged. A mathematical model incorporating ventricular geometry and valve dynamics now enables HDF assessment without direct blood-flow velocity measurements [9].

In diastole, IVPDs reflect ventricular relaxation, compliance, and filling pressure. Apical suction begins at the apex in late systole and propagates toward the base, enabling rapid filling at low pressure [10–12]. Regional IVPDs arise from inertial and convective forces shaped by intraventricular flow, LV geometry, and myocardial relaxation [13, 14]. Aging increases filling pressures while reducing relaxation, which is partly influenced by backward wave dynamics [13].

Noninvasive tools for regional pressure gradients include four-dimensional (4D) flow magnetic resonance imaging, the current reference standard [15, 16], and vector flow mapping (VFM), which reconstructs two-dimensional flow vectors using Doppler and myocardial motion data to provide practical clinical assessment of diastolic IVPDs [17, 18].

Impaired wall motion after myocardial infarction (MI) reduces apical suction [19], disrupting the normal base-to-apex filling gradient [20]. Left ventricular aneurysm (LVA), a mechanical complication of MI, markedly reduces apical suction. We previously reported distinct HDF patterns in patients with LVA with diminished or absent E-waves [21]. In this study, we hypothesized that LVA, a mechanical complication of MI, could decrease apical suction. Furthermore, we investigated the relationship between the IVPDs, as analyzed by VFM, and the pressure difference (PD) from the LA to the LV, as measured through catheter pressure sensors.

To date, no study has validated VFM-derived IVPD against true simultaneous LA–LV pressure measurements using a pressure-sensor wire. The temporal onset of apical suction relative to S2 has not been clinically characterized. By combining VFM, phonocardiography (PCG), and simultaneous dual-chamber pressure recordings, our study aims to provide the first integrated physiological characterization of apical suction and its pathological loss in LVA.

## Materials and methods

### Study design, participants, and ethical approval

This single-center, observational, case–control study did not involve any intervention or treatment allocation. The Ethics Committee of the Dokkyo Medical University Nikko Medical Center approved the study protocol (Ethical License Number Nikko 21 − 006). This study was conducted in accordance with the principles of the Declaration of Helsinki and the Ethics Guidelines for Clinical Research of the Ministry of Health, Labour and Welfare of Japan. Written informed consent was obtained from all patients undergoing cardiac catheterization. The healthy control group provided informed consent through an opt-out process.

We selected 8,361 patients who underwent echocardiography between November 2021 and March 2024. Among these, 181 patients had a left ventricular apical aneurysm. After applying predefined eligibility criteria (sinus rhythm, absence of moderate-to-severe valvular heart disease or decompensated heart failure, and no prior coronary artery bypass graft surgery) and duplicate examinations (*n* = 70), 103 patients with anterior MI with LVA were identified. Of these, 43 patients underwent diagnostic cardiac catheterization, 18 provided written informed consent, and 15 with catheterization and echocardiographic data were included in the final LVA group. During the same period, patients with IHD without LVA who underwent echocardiography and coronary angiography were screened, and 11 patients who provided written informed consent and had complete data were included in the IHD group. A flow diagram summarizing the patient selection process is provided in Supplementary Figure S1. Ischemic heart disease was defined as a history of angina pectoris and MI. On echocardiography, LVA was defined as a well-demarcated area of dyskinesis or akinetic myocardium with outward bulging at the LV apex (apical aneurysm) during systole. In addition, 11 outpatients without IHD who underwent routine echocardiography during the study period were assigned to the H group (*n* = 11). Coronary angiography and invasive pressure measurements were not performed in the H group. Healthy controls were included to provide reference ranges for VFM-derived IVPDs. No inferential statistical comparison with invasive data was intended. The diagnosis was confirmed by two experienced echocardiography specialists. For safety and logistical reasons, cardiac catheterization and echocardiography could not be performed simultaneously. Invasive measurement of the left atrial-left ventricular pressure gradient (ICPD) was performed on the same day whenever possible, or within a tolerance window of ± 4 days, alongside transthoracic echocardiography and VFM analysis. To minimize physiological variability, patients underwent all assessments under stable clinical conditions. No changes were made to the medication or other factors in any patient. Hemodynamic-modifying medications, volume status, and heart failure therapy remained unchanged during the ± 4-day assessment window.

Echocardiography was performed using an ALOKA ARIETTA 850 ultrasound machine (FUJIFILM Healthcare Corporation, Tokyo, Japan) with an S121 probe at a transducer frequency of 1–5 MHz and a depth of 7–9 cm. Whenever possible, echocardiographic examinations were conducted on the same day as coronary angiography; if this was not feasible, they were performed within 3 days of the catheterization. Three experienced sonographers (each with > 10 years of experience in echocardiography) performed all of the acquisitions and VFM analyses. The supine or left lateral recumbent position was used for cardiac image acquisition. Apical or parasternal 2D echocardiograms were obtained by a 90-degree sector scan. A PCG was positioned on the upper sternum to accurately identify the timing of the second heart sound (S2), which was used to define the end of the systolic phase. During the routine echocardiographic examination, VFM images were obtained sequentially; moreover, after the examination, VFM analysis was performed offline. Images for VFM analysis were obtained from apical three-chamber view (3CV), 4CV, and 2CV images, covering the entire LV and LA. These images were configured to display blood flow color images in the LV and LA lumen as the heart contracted and dilated. All images for the VFM analysis were recorded at a fixed frame rate of 48 frames/s. In late systole and early diastole, the IVPD and subsequent intracardiac pressure difference (ICPD; defined as simultaneous LA–LV pressure difference after mitral valve opening) for all patients were analyzed offline frame-by-frame using DAS-RS1 software (version 6.0.0, Fujifilm Healthcare Corporation, Tokyo, Japan) by a single experienced analyst to minimize observer-dependent variability.

In patients with IHD undergoing coronary angiography, the aortic pressure waveform was recorded to identify the position of the dicrotic notch (S2 timing), after which the catheter was advanced into the LV. A pressure wire (PressureWire™ X, Abbott, IL, USA), typically used for fractional flow reserve (FFR) assessment, was employed to simultaneously measure LA–LV pressure gradients in early diastole [22, 23]. After a 30-s stabilization period, the pressures of the pressure sensor and guiding catheter were calibrated and equalized to a reference value of 1.0. The equivalence of LV diastolic pressures obtained from the two systems was visually confirmed, with the upper limit of the pressure waveform display set to 30 mmHg. Following equalization, the wire was retrogradely advanced from the LV into the LA, with the sensor positioned in the LA and the guiding catheter tip placed in the LV. The peak simultaneous pressure difference between the LA and LV (ICPD_Catheter_) was determined by careful waveform inspection in early diastole and averaged over three consecutive heartbeats. This retrograde catheterization technique for simultaneous measurement of LA and LV pressures is a novel and technically demanding method; therefore, all procedures were performed by a single experienced operator.

## Image analysis

**Fig. 1:**
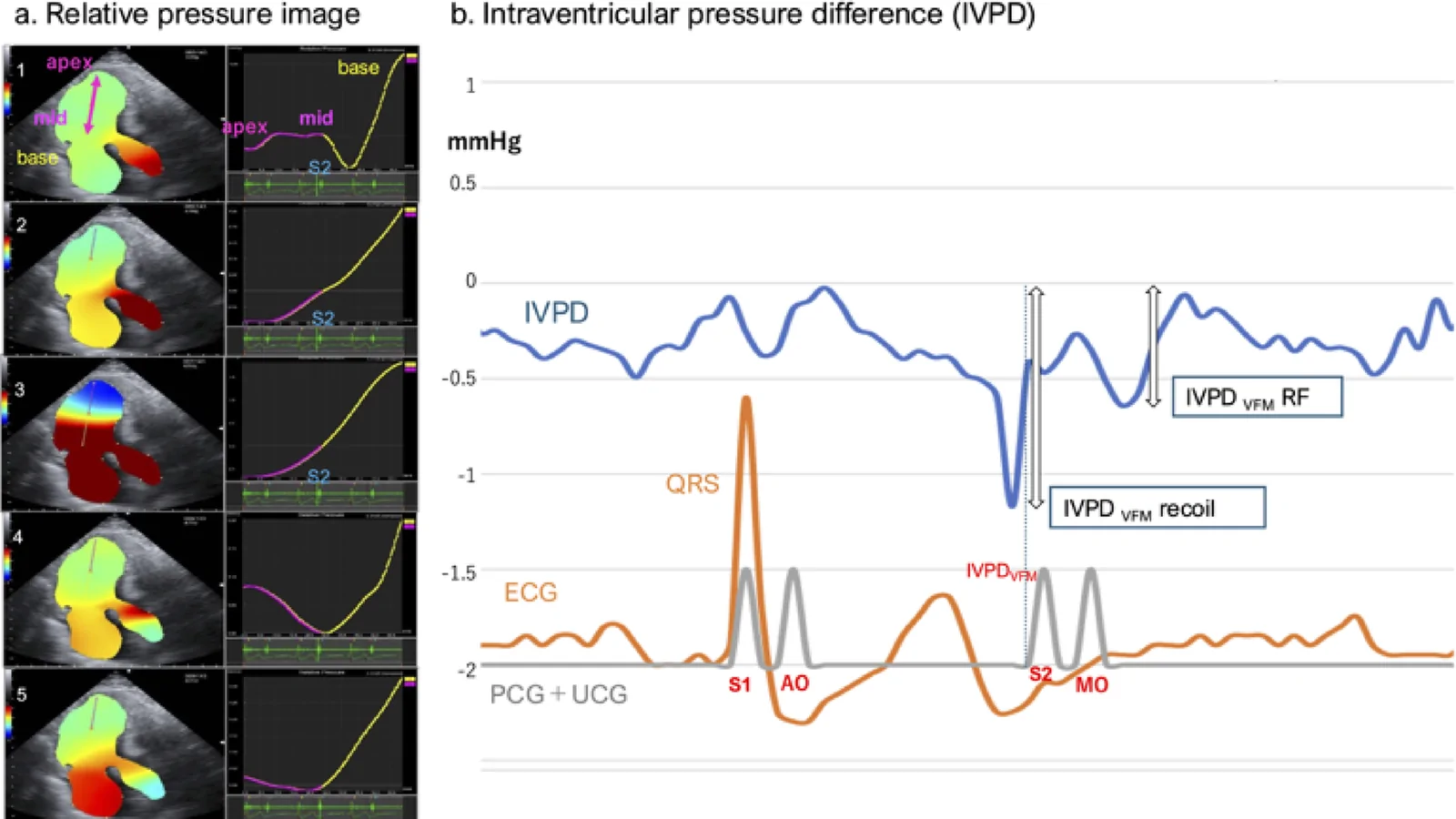
Intraventricular pressure difference (IVPD) from the left ventricular (LV) apex to the base during late systole to early diastole in a healthy male participant, together with its temporal analysis a. In late systole, the relative pressure at the apex was lower than at the base, represented by cold blue colors, indicating the presence of suction (a2, a3) b. Using vector flow mapping (VFM), the intersection of the IVPD curve with the zero line was defined as the onset of apical suction. The peak relative pressure during the recoil period (IVPD_VFM_ recoil) and during early filling immediately after mitral valve opening (IVPD_VFM_ RF) were determined. Each parameter was measured over three consecutive beats for each participant, and the average value was calculated. These parameters, IVPD_VFM_ recoil and IVPD_VFM_ RF are indicated in the figure with white double-headed arrows. AO: Aortic valve opening, MO: Mitral valve opening, PCG: Phonocardiogram, S1: First heart sound, S2: Second heart sound

Relative pressure imaging is a novel, noninvasive method that uses VFM for assessing the intracardiac pressure distribution [18, 24–28]. The VFM intraventricular flow velocity vectors were calculated by solving the continuity equation using the flow velocity obtained from color Doppler and the wall velocity obtained from speckle tracking echocardiography. The velocity data were then converted into a relative pressure distribution using the momentum equations of fluid motion (i.e., Navier–Stokes equations). The VFM was analyzed and the IVPD was plotted along a sampling line from the apex to the base in the LV from late systole to early diastole. A sampling line was drawn from the LV apex to the mid-septal point parallel to the long axis, based on anatomical landmarks, to ensure consistency across studies. In late systole, the relative pressure at the apex was lower than that at the mid LV, represented by cold blue colors, indicating the presence of suction (Figs. 1a2, 1a3). The intersection of the IVPD curve with the zero line was defined as the onset of apical suction using VFM. The peak relative pressures during the recoil period (IVPD_VFM_ recoil) and early filling (IVPD_VFM_ RF) were determined. Both parameters were measured over three consecutive beats for each participant, and the average value was calculated, denoted as IVPD_VFM_ recoil and IVPD_VFM_ RF, respectively. We propose that the IVPD_VFM_ recoil indicates apical suction. We developed a novel method for analyzing VFM and measuring HDF from late systole to early diastole.

### Statistical analysis

Continuous variables are presented as the mean ± standard deviation (SD), and categorical variables are presented as numbers and percentages. Comparisons were performed using Student’s t-test, Fisher’s exact test, Welch’s t-test, Mann–Whitney U test, or chi-square test, as appropriate. Pearson’s correlation coefficient was used to assess the relationship between IVPD_VFM_ parameters (IVPD_VFM_ recoil, IVPD_VFM_ RF, and the sum of IVPD_VFM_ recoil and IVPD_VFM_ RF) and either the peak E-wave velocity or ICPD_Catheter_. The correlation analysis was exploratory, and no multiplicity adjustment was applied. All analyses were performed using JMP-J version 14.0 (SAS Institute, Cary, NC, USA) and SPSS Statistics version 30.0 (IBM Corp., Armonk, NY, USA). A two-tailed P-value of < 0.05 was considered statistically significant.

## Results

A total of 37 participants were included, including 26 with IHD. The patients were further subdivided into patients with LVA (LVA; *n* = 15) and those without LVA (IHD without LVA [IHD group]; *n* = 11). Table 1 summarizes the baseline characteristics. The IHD group comprised three patients with old MI, six patients with stable angina, and two patients with vasospastic angina pectoris. All patients in the LVA group had old anterior MI. The H group included patients with no history of angina or MI (*n* = 11). The baseline characteristics of the H group are summarized in Supplementary Table S1.

**Table 1:** Baseline clinical characteristics

Variable	IHD Group (*n* = 11)	LVA Group (*n* = 15)	*P*-value
*Demographic data*			
Age, years	75 ± 8	71 ± 9	0.25
Male sex, n (%)	6 (55%)	7 (47%)	0.69
*Clinical history*			
Hypertension, n (%)	10 (91%)	14 (93%)	0.82
Dyslipidemia, n (%)	4 (36%)	15 (100%)	0.03
Diabetes mellitus, n (%)	8 (73%)	6 (40%)	0.85
Chronic kidney disease	2 (18%)	2 (13%)	0.74
Heart failure (AHA/ACC stage C), n (%)	3 (27%)	6 (40%)	0.50
Previous cerebral infarction, n (%)	1 (9%)	2 (13%)	0.74
Peripheral artery disease, n (%)	2 (18%)	2 (13%)	0.74
*Ischemic heart disease*			
Stable angina pectoris, n (%)	6 (55%)	6 (40%)	0.46
• Vasospastic angina pectoris	2	0	
Previous MI, n (%)	5 (45%)	15 (100%)	0.01
• Anterior wall MI, n	3	15	
• Inferior wall MI, n	1	0	
• Lateral wall MI, n	1	0	
Prior PCI, n (%)	6 (55%)	15 (100%)	< 0.01
Prior CABG, n (%)	0 (0%)	0 (0%)	
*Coronary angiographic findings*			
*Number of diseased vessels, n (%)*			
0-vessel disease	8 (73%)	5 (33%)	
1-vessel disease	2 (18%)	6 (40%)	
2-vessel disease	0 (0%)	3 (20%)	
3-vessel disease	1 (9%)	1 (7%)	

*ACC*: American college of cardiology, *AHA*: American heart association, *MI*: Myocardial infarction, *IHD group*: Patients with ischemic heart disease without left ventricular aneurysm (LVA), *LVA group*: Patients with ischemic heart disease with LVA

Echocardiography showed a significantly higher left ventricular end-diastolic volume (LVEDV) and left ventricular end-systolic volume (LVESV) in the LVA group, whereas the E/A ratio and E/e′ (an index of LV filling pressure) did not differ between the IHD and LVA groups (Table 2). No significant differences in blood pressure or heart rate were observed between the groups. No significant differences in echocardiography parameters were found between the H and IHD groups (Supplementary Table S2).

**Table 2:** Comparison of echocardiographic, vital, and cardiac catheterization data

Variables	IHD Group (*n* = 11)	LVA Group (*n* = 15)	*P*-value
LVDd (mm)	43 ± 3	50 ± 7	0.01
LVDs (mm)	27 ± 4	34 ± 8	0.03
LVEDV (mL)	80 ± 9	122 ± 48	< 0.01
LVESV (mL)	31 ± 7	59 ± 31	< 0.01
EF (%)	60 ± 11	52 ± 9	0.06
E-wave (m/sec)	0.7 ± 0.2	0.7 ± 0.2	0.92
E/A	0.9 ± 0.4	0.8 ± 0.4	0.68
E/e’	12 ± 5	14 ± 5	0.42
e’ (cm/sec)	5.8 ± 1.3	5.4 ± 1.9	0.51
LAVI (mL/m^2^)	30 ± 18	37 ± 13	0.05
LAEI (%)	104 ± 54	101 ± 39	0.90
VMT score: n (%) 0 1 2	7 (64%) 3 (27%) 1 (9%)	9 (60%) 4 (27%) 2 (13%)	0.945*
IVPD_VFM_ recoil (mmHg)	2.5 ± 1.1	1.0 ± 0.7	< 0.01
IVPD_VFM_ RF (mmHg)	2.1 ± 1.7	1.7 ± 0.9	0.89
IVPD_VFM_ recoil + RF (mmHg)	4.6 ± 1.9	2.7 ± 1.2	< 0.01
Systolic BP (mmHg)	122 ± 16	132 ± 24	0.26
Diastolic BP (mmHg)	73 ± 14	76 ± 14	0.44
Heart rate (beats/sec)	67 ± 7	73 ± 15	0.38
LVEDP (mmHg)	12.4 ± 5.1	17.3 ± 8.5	0.09
Peak LA–LV pressure difference (ICPD_Catheter_) (mmHg)	8.0 ± 2.7	6.5 ± 1.9	0.12

*IHD* group: Patients with ischemic heart disease without left ventricular aneurysm (LVA), *LVA group*: Patients with ischemic heart disease with LVA, *LVDd*: Left ventricular end-diastolic diameter, *LVD*: Left ventricular end-systolic diameter, *EF*: Ejection fraction, *LVEDV*: Left ventricular end-diastolic volume, *LVESV*: Left ventricular end-systolic volume, *E/A*: Ratio of the early (E) to late (A) ventricular filling velocities, *E/e’*: Ratio of early diastolic mitral inflow velocity to early diastolic mitral annular velocity, *LAVI*: Left atrial volume index, *LAEI*: Left atrial expansion index, *IVPD*_*VFM*_ *recoil*: Intraventricular pressure difference by VFM during LV recoil in end systole, *IVPD*_*VFM*_ *RF*: IVPD by VFM during rapid filling, *BP*: Blood pressure, *LVEDP*: Left ventricular end-diastolic pressure, *ICPD*_*Catheter*_: Peak pressure gradient between the left atrium and left ventricle during early diastole, obtained by simultaneous pressure measurement in cardiac catheterization, *VMT score*: Visually assessed time difference between the mitral valve and tricuspid valve opening score

**Fig. 2:**
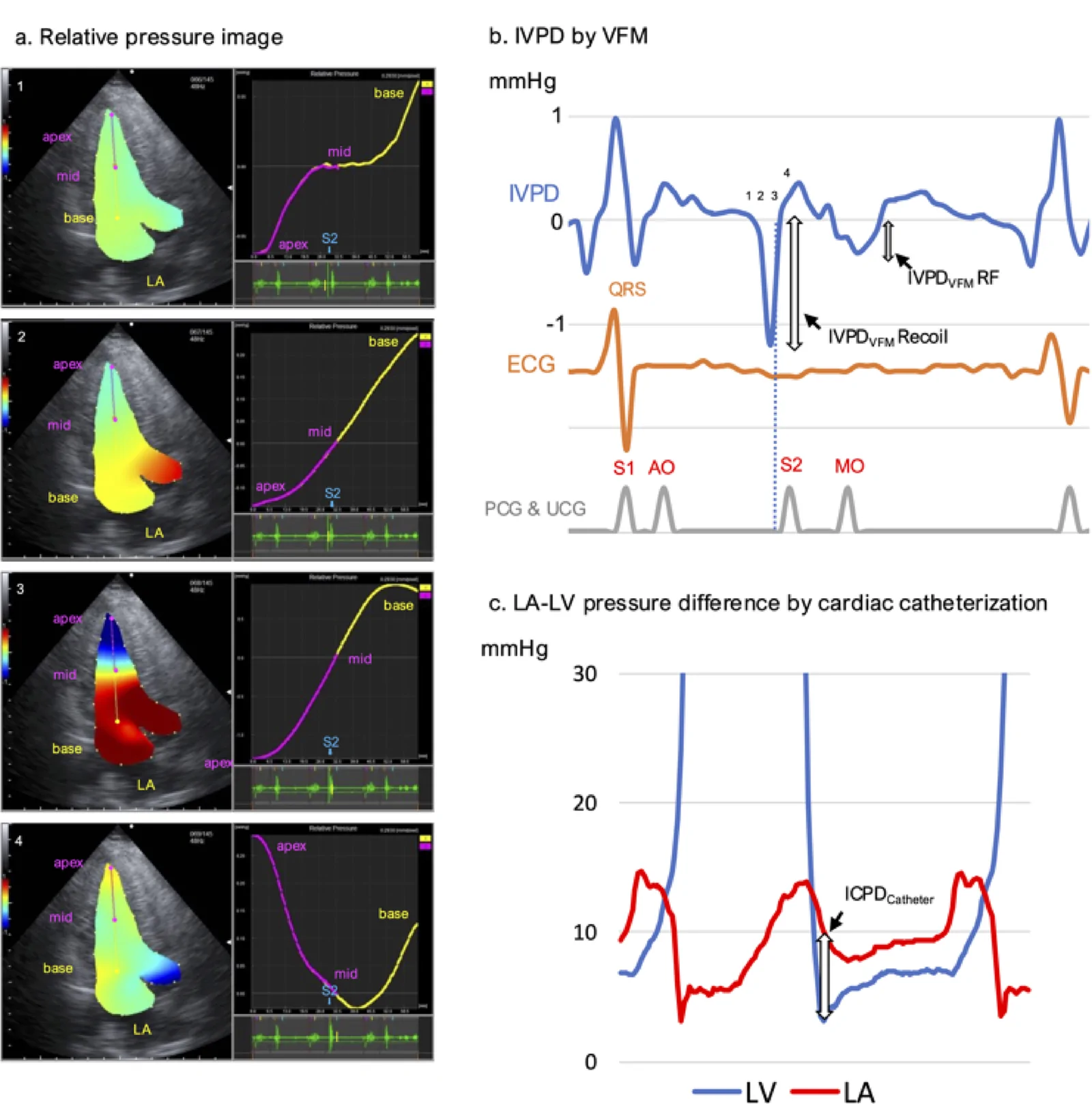
Representative patient in the group of ischemic heart disease (IHD) without left ventricular aneurysm (IHD group) with a left ventricular end-diastolic pressure (LVEDP) of 14 mmHg a. Intraventricular pressure difference (IVPD) images from late systole to early diastole are shown in the left panels **a**. The corresponding apex-to-mid and apex-to-base relative pressure curves are displayed on the right **b**. Warm colors indicate higher pressure, whereas cold colors indicate lower pressure relative to the reference point. Four frames were extracted relative to the second heart sound (S2): the two preceding frames (1, 2), the frame at S2 (3), and the frame immediately after S2 (4) b. Apical suction analyzed by vector flow mapping (VFM) using the frame-by-frame method on echocardiography. The blue line represents the IVPD, the orange line represents the electrocardiogram (ECG), and the gray line represents the phonocardiogram (PCG) and ultrasound cardiogram (UCG). Points 1–4 on the IVPD curve correspond to the frames in panel A. VFM analysis enabled extraction of the relative intraventricular pressure difference due to apical recoil from late systole to isovolumic relaxation (IVPD_VFM_ recoil), as well as the IVPD during rapid filling in early diastole (IVPD_VFM_ RF). These parameters are indicated in the figure by white double-headed arrows c. Simultaneous left atrial (LA)-LV pressure difference measured during diastole with a pressure sensor wire during cardiac catheterization. The blue line indicates LV pressure, and the red line indicates LA pressure. The peak LA–LV pressure difference in early diastole was measured as intracardiac pressure difference (ICPD_Catheter_). The peak pressure difference is also indicated with white double-headed arrows. It should be noted that the IVPD_VFM_ and ICPD_Catheter_ were obtained on the same day but not simultaneously

**Fig. 3:**
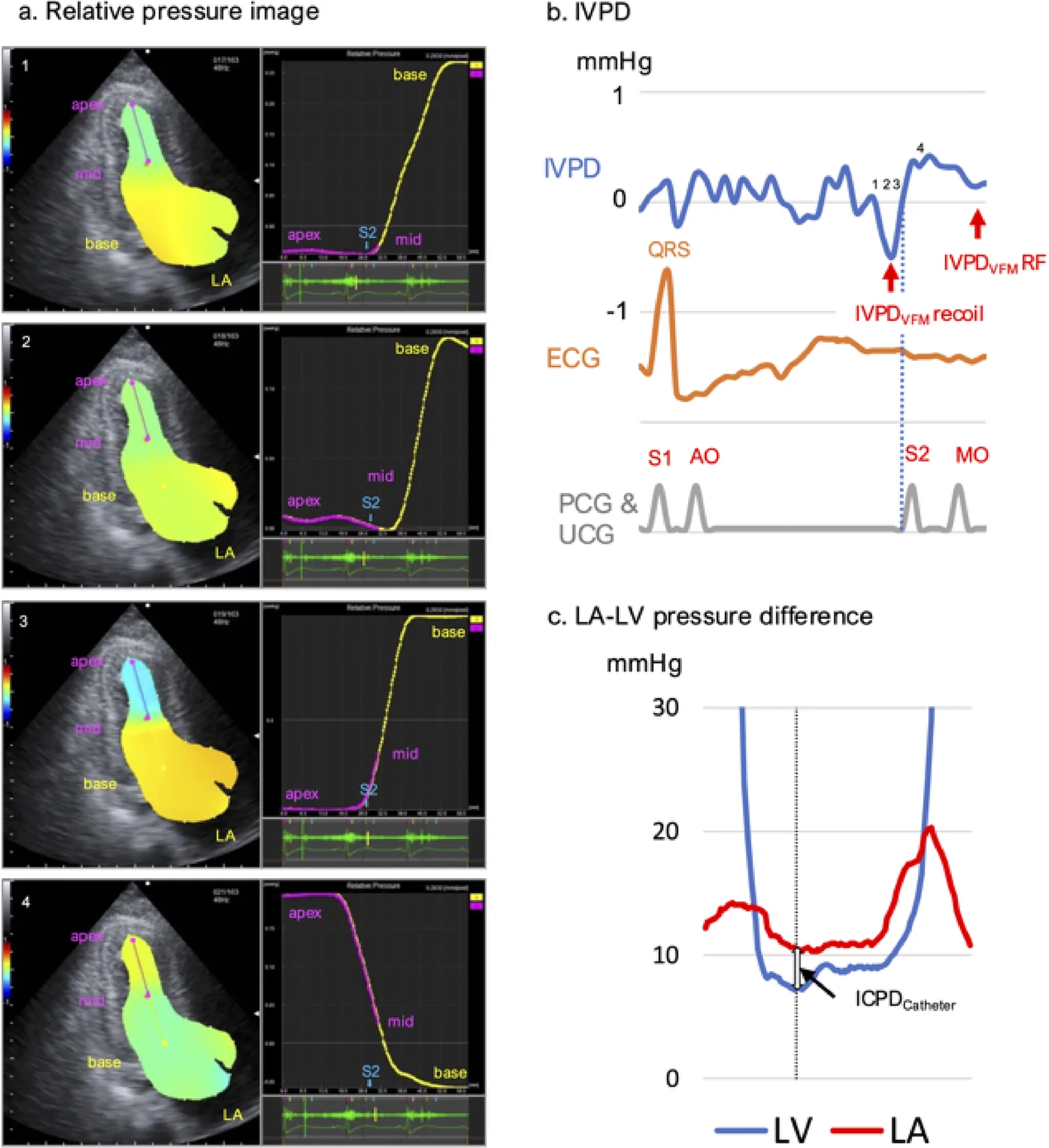
Representative patient in the group with myocardial infarction and left ventricle aneurysm (LVA group) with a left ventricular end-diastolic pressure (LVEDP) of 18 mmHg a. Intraventricular pressure difference (IVPD) images during late systole to early diastole. Warm colors indicate higher, and cold colors indicate lower pressure relative to the reference point. Relative pressure images were extracted at two frames preceding the second heart sound (S2) (1, 2), the frame at S2 (3), and the frame immediately after S2 (4), using S2 as the reference point b. The apex-to-base IVPD curves during late systole to early diastole. The blue line indicates the IVPD, the orange line indicates ECG, and the gray line indicates the phonocardiogram (PCG) and ultrasound cardiogram (UCG). Points 1–4 on the IVPD curve correspond to the frames shown in panel A. The peak IVPD during LV recoil was defined as IVPD_VFM_ recoil. The depth of IVPD_VFM_ recoil is shallower than that in Fig. 1b (ischemic heart disease without LVA) c. The simultaneous left atrial (LA)-LV pressure difference was measured using a pressure sensor wire in the LA and JR guide catheter in the LV. The peak of the LA–LV simultaneous pressure difference in early diastole was measured as the peak pressure difference (ICPD_Catheter_). Notably, LV and LA pressures were elevated in early diastole and in end-diastole, compared with Fig. 2c (an IHD case without LV aneurysm)

**Fig. 4:**
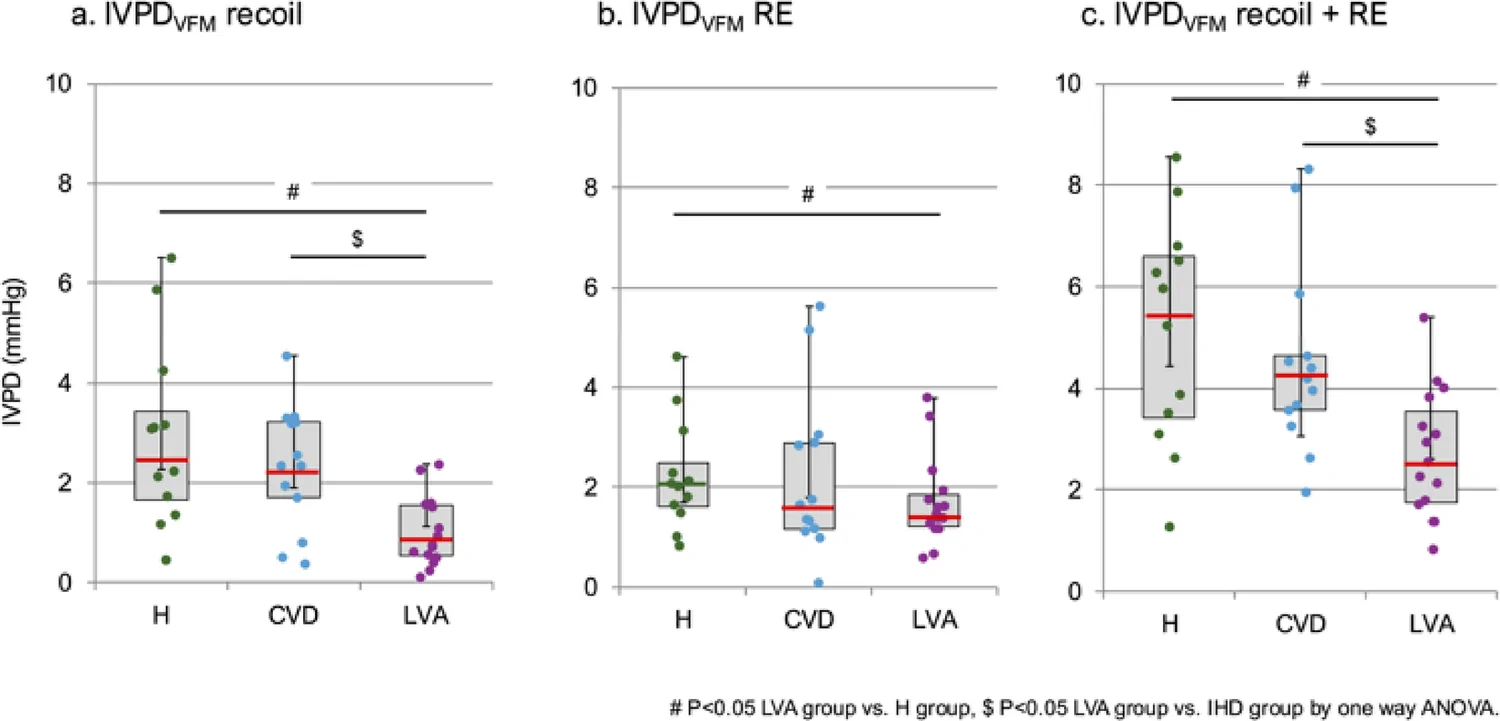
Distribution of intraventricular pressure difference (IVPD) between the LV apex and LV base, measured by vector flow mapping (VFM), across three physiological phases: recoil, rapid filling, and the sum of recoil and rapid filling. Data are shown for patients with ischemic heart disease without LV aneurysm (IHD group, n = 11) and for those with myocardial infarction and LV aneurysm (LVA group, n = 15) Box-and-whisker plots display the median (central line), mean (cross), interquartile range (IQR; box), and the minimum and maximum values within 1.5 × IQR (whiskers). Individual data points are plotted as scattered dots. Statistical comparisons between groups were performed using Student’s t tests, and uncorrected P-values are shown above the brackets IVPD during LV recoil in late systole and the isovolumic relaxation period (IVPD_VFM_ recoil) was significantly lower in the LVA group than in the IHD group IVPD during early-diastolic rapid filling (IVPD_VFM_ RF​) did not differ significantly between the two groups The combined IVPD_VFM_ recoil​ and IVPD_VFM_ RF​, representing the driving force of the transmitral E-wave, was significantly reduced in the LVA group compared with the IHD group

Figure 1 shows the representative relative pressure maps and apex-to-base IVPD curves for the H group. Lower apical relative pressure (cold colors) indicated apical suction in late systole, whereas basal suction characterized early mitral valve opening. The peak relative pressures during the recoil period (IVPD_VFM_ recoil) and early filling (IVPD_VFM_ RF) were quantified by offline analysis and averaged over three consecutive beats. The recoil and RF values in the H group indicated effective apical and basal suction, respectively. In the IHD group (Fig. 2), apical suction was evident in late systole, with lower apical relative pressure before S2 (Fig. 2a1–3), and early filling was accompanied by basal suction (Fig. 2a4). In contrast, in the LVA group (Fig. 3), although early diastolic inflow from the LA to LV was observed (Fig. 3a4), the color change was minimal, indicating a reduced relative pressure difference. The IVPD_VFM_ recoil was significantly lower in the LVA group than in the IHD group, suggesting reduced apical suction (Fig. 4a). In contrast, the IVPD_VFM_​ RF was not different between the two groups (Fig. 4b). Consequently, the sum of IVPD_VFM_ recoil and IVPD_VFM_​ RF, reflecting the overall driving force, was significantly lower in the LVA group than in the IHD group (Fig. 4c).

**Fig. 5:**
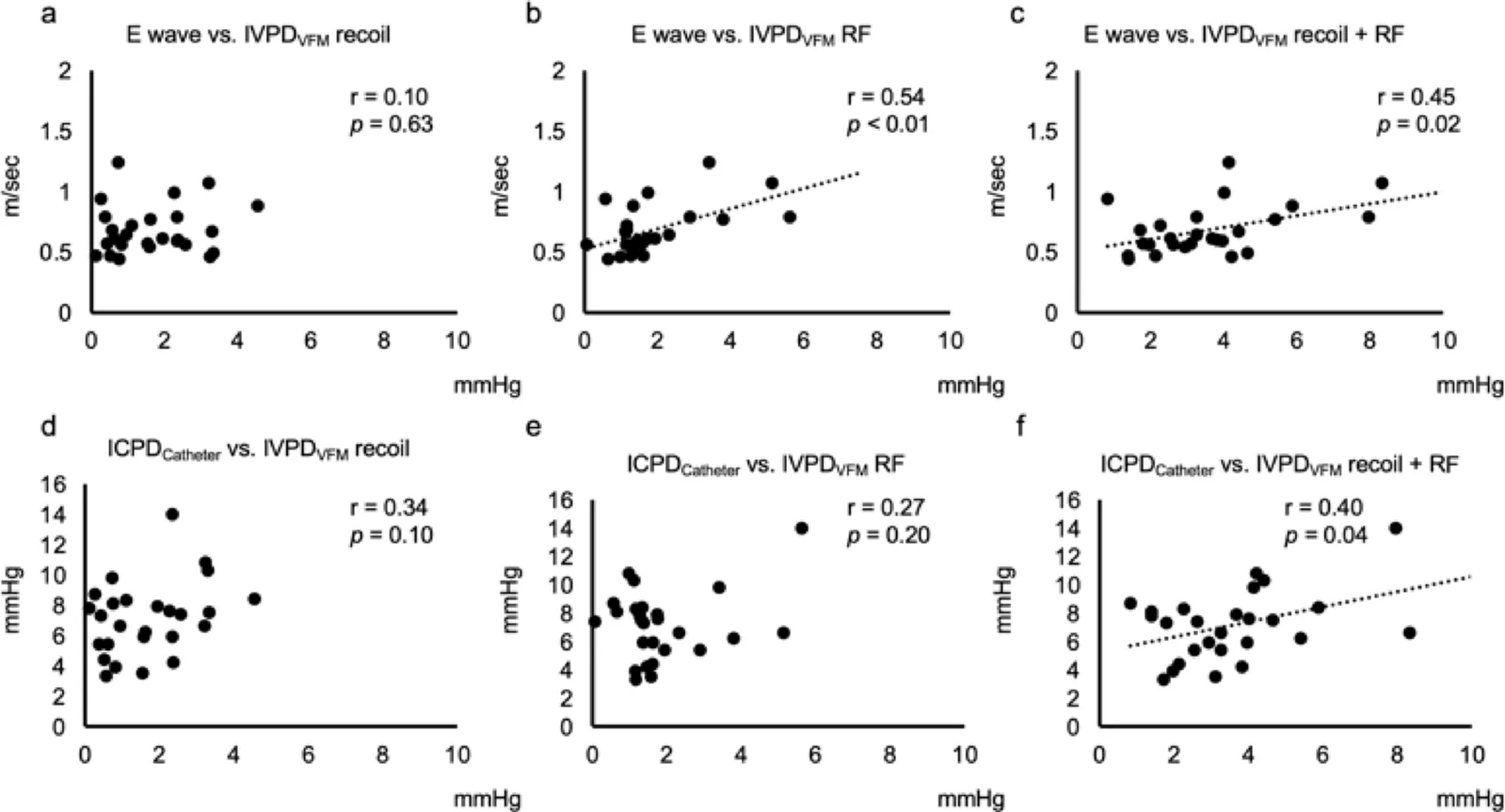
Correlation between measured parameters Correlation between transmitral E-wave peak velocity (m/s) and IVPD_VFM_ recoil (a), IVPD_VFM_ RF (b), and the sum of IVPD_VFM_ recoil + IVPD_VFM_ RF (c), as well as between the early diastolic peak LA–LV pressure difference measured by simultaneous left atrial and left ventricular catheterization (ICPD_Catheter_) and IVPD_VFM_ recoil (d), IVPD_VFM_ RF (e), and IVPD_VFM_ recoil + IVPD_VFM_ RF (f). IVPD_VFM_ recoil reflecting apical suction beginning in late systole, showed no significant correlation with either the E-wave or ICPD_Catheter_ (a, d). In contrast, the IVPD_VFM_ RF demonstrated a significant correlation with the E-wave (*r* = 0.54, *P* < 0.01) (b), and the combined parameter IVPD_VFM_ recoil + IVPD_VFM_ RF was also correlated with the E-wave (*r* = 0.45, *P* = 0.03) (c). A significant correlation was observed between the sum of IVPD_VFM_ recoil + IVPD_VFM_ RF and ICPD_Catheter_ (*r* = 0.40, *P* < 0.05). Uncorrected P-values are shown in these figures

Correlation analyses between IVPD_VFM_ parameters and either the transmitral E-wave peak velocity or ICPD_Catheter_ are shown in Fig. 5a–f. The IVPD_VFM_ recoil, reflecting apical suction beginning in late systole, showed no significant correlation with either the transmitral E-wave peak velocity or ICPD_Catheter_ (Fig. 5a and d). In contrast, IVPD_VFM_ RF and the combined IVPD_VFM_ recoil + RF parameter correlated significantly with the E-wave (*r* = 0.54, *P* < 0.01 and *r* = 0.45, *P* = 0.03, respectively; Fig. 5b and c). A significant correlation was observed between the combined IVPD_VFM_ parameter and ICPD_Catheter_ (*r* = 0.40, *P* < 0.05; Fig. 5f). A trend toward correlation was observed between the echocardiographic E/A ratio and LVEDP in the IHD group (*r* = 0.55, *P* = 0.07). In contrast, no such correlation was observed in the LVA group (*r* = 0.02, *P* = 0.94) (Supplementary Figure S2). The VFM-derived IVPD correlated with mitral annular e′, supporting its relevance to diastolic relaxation (Supplementary Figure S3). Supplementary Figure S4 presents the additional correlations between the VFM-derived IVPD parameters and other echocardiographic indices. Among these parameters, LVESV showed a modest inverse correlation with IVPD_VFM_ recoil (*r* = − 0.49, *P* = 0.01), whereas correlations with other parameters were not significant.

Additionally, serial echocardiographic and catheterization data were obtained from two patients from the LVA group (case 1: a 52-year-old man; case 2: a 78-year-old woman) during hospitalization for worsening heart failure. During worsening heart failure, both patients had markedly elevated LVEDP and abnormal mitral valve and tricuspid valve opening (VMT) scores; however, the E/A ratio was < 1.0 in both patients, and their IVPD_VFM_ recoil was reduced. After appropriate heart failure treatment, LVEDP decreased to 12 and 9 mmHg, respectively. With preload reduction, IVPD_VFM_​ RF increased in both cases, and the combined IVPD_VFM_ recoil + RF increased in both cases. The IVPD_VFM_ recoil increased in case 1 but remained unchanged in case 2 (Table 3).

**Table 3:** Serial changes in echocardiographic and cardiac catheterization data of the two patients in the left ventricle aneurysm (LVA) group who were hospitalized for heart failure

Parameter	Case 1	Case 1 (after 5 months)	Case 2	Case 2 (after 14 months)
Systolic BP (mmHg)	125	125	188	98
Diastolic BP (mmHg)	82	74	104	51
Heart rate (beats/sec)	93	84	70	59
NT-proBNP (pg/mL)	3,665	412	4,019	432
LVDd (mm)	68	60	48	38
LVDs (mm)	57	50	28	25
EDV (mL)	277	263	117	95
ESV (mL)	143	164	60	55
EF (%)	49	38	49	42
E-wave (m/s)	0.94	0.52	0.64	0.53
E/A	0.6	0.74	0.6	0,63
DT (msec)	120	240	190	248
E/e’	15.7	10.3	14.9	7.65
LAVI (mL/m^2^)	42	33	44	33
LAEI (%)	99	68	88	65
VMT score (point)	2	0	1	0
IVPD_VFM_ recoil (mmHg)	0.26	0.50	0.94	0.84
IVPD_VFM_ RF (mmHg)	0.57	0.98	2.33	4.49
IVPD_VFM_ recoil + RF (mmHg)	0.82	1.48	3.26	5.33
LVEDP (mmHg)	32.8	11.5	24.6	8.7
Peak LA–LV pressure difference (ICPD_Catheter_) (mmHg)	8.7	6.6	6.6	4.4

*BP*: Blood pressure, *NT-proBNP*: N-terminal pro-brain natriuretic peptide, *LVDd*: Left ventricular end-diastolic diameter, *LVD*: Left ventricular end-systolic diameter, *LVEDV*: Left ventricular end-diastolic volume, *LVESV*: Left ventricular end-systolic volume, *EF*: Ejection fraction, *E/A*: Ratio of the early (E) to late (A) ventricular filling velocities, *DT*: Deceleration time, *E/e’*: Ratio of early diastolic mitral inflow velocity to early diastolic mitral annular velocity, *LAVI*: Left atrial volume index, *LAEI*: Left atrial expansion index, *IVPD*_*VFM*_ recoil: Intraventricular pressure difference by VFM during LV recoil in end systole, *IVPD*_*VFM*_ *RF*: IVPD by VFM during rapid filling, *ICPD*_*Catheter*_: Peak pressure gradient between the left atrium and left ventricle during early diastole, obtained by simultaneous pressure measurement in cardiac catheterization, *LVEDP*: Left ventricular end-diastolic pressure, *VMT score*: Visually assessed time difference between the mitral valve and tricuspid valve opening score

## Discussion

VFM combined with PCG was used to evaluate IVPD from late systole to early diastole, which was validated against simultaneous LA–LV pressure recordings obtained using a pressure-sensor wire. The principal finding was that patients with LVA exhibited markedly reduced apical suction, reflected by significantly lower IVPD_VFM_ recoil and the combined IVPD_VFM_ recoil + RF, compared with patients with IHD and without LVA (IHD group). This impaired suction was accompanied by reduced LA–LV driving pressure during early filling. Because normative VFM-derived IVPD data have not yet been established, we also included healthy participants to establish reference values and clarify the physiological timing of apical suction from late systole to early diastole. Thus, we aimed to clarify the pathophysiological role of apical suction impairment in LVA by validating VFM-derived IVPD against invasive catheter measurements.

### Impaired apical suction in patients with LVA

In patients with LVA, the IVPD_VFM_ recoil and combined IVPD_VFM_ recoil + RF were significantly reduced. This is consistent with prior reports showing that regional wall-motion abnormalities and geometric distortion following MI impair diastolic suction. The reduction in IVPD_VFM_​ recoil is expected to elevate LV pressure in early diastole and delay mitral valve opening [24, 25]. Further studies with larger cohorts are required to clarify these relationships.

## Real-time IVPD_VFM_ analysis using PCG

Real-time IVPD_VFM_ has been previously validated [17, 18, 26–28]. The systolic phase was defined as the time from the onset of the QRS complex on echocardiography to S2. In healthy participants, apical suction begins in late systole before S2, generating a base-to-apex pressure gradient that facilitates efficient early diastolic filling. By integrating PCG into the echocardiographic system, we were able to detect the precise timing of S2 and thereby define the onset of apical suction as IVPD_VFM_ recoil. Our results demonstrated that apical suction begins approximately 60–70 ms before S2, which is consistent with previous experimental findings [5, 12]. This indicates that blood flow vector changes precede measurable pressure differences, suggesting a partial overlap between the systolic and diastolic phases. Although limited by a frame rate of 48 fps, this approach provided temporal resolution not previously achieved in clinical VFM studies. The analysis was performed using a frame-by-frame method, which inherently restricts the temporal resolution. Sørensen et al. reported excellent agreement between blood speckle tracking (BST)-derived IVPD and invasive pressure measurements, emphasizing that frame rates above 60 fps are required for accurate IVPD estimation [20].

## Relationship between transmitral flow and invasive pressure

The IVPD_VFM_ RF, reflecting basal suction after mitral valve opening, correlated significantly with transmitral E-wave velocity, whereas the IVPD_VFM_ recoil did not. The combined parameter (IVPD_VFM_ recoil + RF) showed a moderate correlation with the E-wave and a significant correlation with ICPD_Catheter_, supporting its potential utility as an integrated index of the diastolic driving force. The sum of IVPD_VFM_ recoil and RF represents the total diastolic restoring and suction forces, reflecting the integrated LV relaxation capacity. The IVPD_VFM_ RF has a greater impact on E-wave compared with recoil, possibly because RF represents the basal-to-apical dynamic pressure gradient acting directly during early rapid filling. However, simultaneous invasive LA–LV pressure reflects the integrated diastolic driving forces beyond valve-opening–dependent gradients; therefore, dysfunction might manifest most clearly in the combined index. Courtois et al. first demonstrated the importance of regional IVPDs in driving early diastolic filling, showing that transmitral flow velocity is determined by the LA–LV pressure difference and apex-to-base pressure gradients within the LV [6]. Apical suction has since been recognized as a key physiological mechanism underlying rapid ventricular filling and diastolic function. Collectively, these observations underscore the limitation of relying solely on transmitral flow velocity as a surrogate for LV filling pressure, particularly in pathological conditions such as LVA [21].

The standard indices reflecting LV diastolic function, such as transmitral E-wave velocity or E/A on echocardiography might be influenced by hemodynamic status [29, 30]. Peverill RE et al. reported that the E-wave is influenced by the LA–LV pressure gradient and various other factors, including LV relaxation, heart rate, and blood pressure [31]. Furthermore, when the LV is not sufficiently relaxed or the wall stiffness is increased, the E-wave inflow velocity does not rise adequately even in the presence of an LA–LV pressure gradient, resulting in flow limitation despite a high pressure gradient [32, 33]. In our previous case series of four patients with a large LVA [34], we showed a possible explanation for the impaired relaxation pattern (E/A < 1) observed despite the high PAWP during right heart catheterization in patients with LVA. Similarly, in a previously reported study, patients with MI and LVA also had a PAWP of > 18 mmHg, even though they presented with an impaired relaxation-type pattern (E/A < 1) [21]. Thus, inferring LVEDP from the E/A ratio alone could be challenging under abnormal circumstances, such as the presence of an LVA [35, 36]. The VFM-derived IVPD may represent the dynamic suction effect rather than the difference in static pressure. This finding strengthens the physiological validity of the IVPD_VFM_ as a noninvasive surrogate of myocardial relaxation, complementing traditional Doppler parameters. In addition, exploratory correlations with other echocardiographic indices showed only a modest inverse association between LVESV and IVPD_VFM_ recoil, whereas no meaningful correlations were observed with e′, E/e′, left atrial volume index, or LV ejection fraction.

### Effects of preload reduction

The two cases with old anterior MI with LVA (LVA group) demonstrated that the response of IVPD components to preload reduction depends on regional myocardial viability. In patients with apical aneurysm, where contractility and suction are absent, IVPD_VFM_ recoil was insensitive to preload changes. In contrast, mid-to-basal LV segments with preserved wall motion exhibited increased IVPD_VFM_​ RF after preload reduction, indicating that regional viability determines the relative contribution of IVPD components to global LV filling. Taken together, these findings support the concept that the presence or absence of viable myocardium determines the contribution of regional IVPDs to global LV filling and may provide mechanistic insight into diastolic adaptation after heart failure therapy in patients with post-infarction LV remodeling.

### Technical aspects

Accurate simultaneous measurement of LA and LV pressures is essential for investigating hemodynamics during late systole and early diastole. In routine clinical practice, LA–LV pressure gradients (e.g., in mitral stenosis) are often estimated by recording the PAWP with a Swan–Ganz catheter in combination with LV pressure from a pigtail catheter. However, this method introduces a time lag between the PAWP and true LA pressure waveform, making it unsuitable for assessing hemodynamic forces during the transition from end-systolic to early diastolic. To address this limitation, we employed a novel technique in which a pressure-sensor wire, typically used for FFR assessment, was advanced retrogradely from the LV into the LA. This technique allowed safe and accurate simultaneous measurement of LA and LV pressures. Although the Brockenbrough technique has also been described for direct LA pressure recording, its risk of cardiac tamponade limits its applicability in clinical research.

### Clinical implications

The VFM-derived IVPD provides noninvasive insight into diastolic sαuction and LA–LV driving pressure, which complements conventional Doppler indices [5, 36]. Impaired apical suction may explain the discrepancy between Doppler-derived filling patterns and invasively measured filling pressures in patients with LVA. Thus, VFM may enhance the physiological assessment of diastolic function in patients with post-infarction LV remodeling.

### Limitations

This was a single-center study with a small sample size. Second, invasive measurements were performed only in the IHD and LVA groups; thus, we primarily aimed to compare these two groups. Healthy controls were included to provide reference values for VFM-derived IVPDs. For ethical reasons, invasive pressure measurements could not be performed in healthy participants. Third, the temporal resolution of VFM (48 fps) limited the precise analysis of suction onset relative to valve events. Fourth, catheter-derived ICPD and VFM-derived IVPD are measured using different principles and timing within the cardiac cycle, which may partly explain the modest correlation between them. Fifth, although every effort was made to perform catheterization and echocardiography on the same day or within a few days, subtle changes in hemodynamic status may still have occurred. Sixth, the IHD group included 3 patients with anterior MI without aneurysm. However, this subgroup was statistically underpowered for independent comparison. Therefore, instead of separately evaluating these cases, they were incorporated into the IHD group. In addition, infarct size was not standardized across MI regions due to the limited number of cases available for each subtype. This heterogeneity is acknowledged as a limitation of the study. Seventh, invasive simultaneous LA–LV pressure is expected to be influenced by both IVPD_VFM_ recoil and RF. We expect that if statistical power increased with a larger number of cases, both IVPD_VFM_ recoil and RF might have individually demonstrated the significant correlation, a possibility that requires validation in future studies. Finally, selection bias may have been introduced because patients in the LVA and IHD groups were selected based on the availability of diagnostic cardiac catheterization, adequate echocardiographic image quality, and complete data. Therefore, the findings should be interpreted as exploratory. Future studies targeting anterior MI populations are necessary to validate these findings and determine their prognostic significance.

## Conclusion

VFM analysis demonstrated that apical suction is markedly attenuated in patients with LVA, leading to reduced LA–LV driving force during early diastole. The correlation between VFM-derived IVPDs and catheter-derived pressure differences suggests that VFM can serve as a practical, noninvasive tool for evaluating impaired diastolic suction in IHD. In particular, the combined IVPD_VFM_ recoil + RF serves as a physiological marker that Doppler indices alone cannot capture. These findings support the clinical utility of VFM for evaluating LV remodeling and diastolic dysfunction after infarction.

## Supplementary Information

Below is the link to the electronic supplementary material.

Supplementary Material 1

## Data Availability

The datasets generated and/or analyzed during the current study are available from the corresponding author on reasonable request.

## References

[1] Pedrizzetti G, La Canna G, Alfieri O, Tonti G (2014) The vortex—an early predictor of cardiovascular outcome? Nat Rev Cardiol 11:545–553 24889521 10.1038/nrcardio.2014.75

[2] Pedrizzetti G, Martiniello AR, Bianchi V, D’Onofrio A, Caso P, Tonti G (2015) Cardiac fluid dynamics anticipates heart adaptation. J Biomech 48:388–391 25529139 10.1016/j.jbiomech.2014.11.049

[3] Eriksson J, Zajac J, Alehagen U, Bolger AF, Ebbers T, Carlhäll CJ (2017) Left ventricular hemodynamic forces as a marker of mechanical dyssynchrony in heart failure patients with left bundle branch block. Sci Rep 7:2971 28592851 10.1038/s41598-017-03089-x PMC5462838

[4] Arvidsson PM, Töger J, Pedrizzetti G, Heiberg E, Borgquist R, Carlsson M, Arheden H (2018) Hemodynamic forces using four-dimensional flow MRI: an independent biomarker of cardiac function in heart failure with left ventricular dyssynchrony? Am J Physiol Heart Circ Physiol 315:H1627–H1639 30216113 10.1152/ajpheart.00112.2018

[5] Vallelonga F, Airale L, Tonti G, Argulian E, Milan A, Narula J, Pedrizzetti G (2021) Introduction to hemodynamic forces analysis: moving into the new frontier of cardiac deformation analysis. J Am Heart Assoc 10:e023417 34889114 10.1161/JAHA.121.023417 PMC9075239

[6] Courtois M, Kovács SJ Jr, Ludbrook PA (1988) Transmitral pressure-flow velocity relation. Importance of regional pressure gradients in the left ventricle during diastole. Circulation 78:661–671 3409502 10.1161/01.cir.78.3.661

[7] Ebbers T, Wigström L, Bolger AF, Engvall J, Karlsson M (2001) Estimation of relative cardiovascular pressures using time-resolved three-dimensional phase contrast MRI. Magn Reson Med 45:872–879 11323814 10.1002/mrm.1116

[8] Pedrizzetti G, Martiniello AR, Bianchi V, D’Onofrio A, Caso P, Tonti G (2016) Changes in electrical activation modify the orientation of left ventricular flow momentum: novel observations using echocardiographic particle image velocimetry. Eur Heart J Cardiovasc Imaging 17:203–209 26060201 10.1093/ehjci/jev137 PMC4882880

[9] Pedrizzetti G (2019) On the computation of hemodynamic forces in the heart chambers. J Biomech 95:109323 31492418 10.1016/j.jbiomech.2019.109323

[10] Sabbah HN, Stein PD (1981) Pressure-diameter relations during early diastole in dogs. Incompatibility with the concept of passive left ventricular filling. Circ Res 48:357–365 7460209 10.1161/01.res.48.3.357

[11] Yellin EL, Hori M, Yoran C, Sonnenblick EH, Gabbay S, Frater RW (1986) Left ventricular relaxation in the filling and nonfilling intact canine heart. Am J Physiol 250:H620–H629 3963218 10.1152/ajpheart.1986.250.4.H620

[12] Smiseth OA, Steine K, Sandbaek G, Stugaard M, Gjolberg T (1998) Mechanics of intraventricular filling: study of LV early diastolic pressure gradients and flow velocities. Am J Physiol 275:H1062–H1069 9724314 10.1152/ajpheart.1998.275.3.H1062

[13] Bello H, Norton GR, Peterson VR, Mmopi KN, Mthembu N, Libhaber CD, Masiu M, Da Silva Fernandes D, Bamaiyi AJ, Peters F, Sareli P, Woodiwiss AJ (2020) Hemodynamic determinants of age versus left ventricular diastolic function relations across the full adult age range. Hypertension 75:1574–1583 32248702 10.1161/HYPERTENSIONAHA.119.14622

[14] Yotti R, Bermejo J, Antoranz JC, Desco MM, Cortina C, Rojo-Alvarez JL, Allué C, Martín L, Moreno M, Serrano JA, Muñoz R, García-Fernández MA (2005) A noninvasive method for assessing impaired diastolic Suction in patients with dilated cardiomyopathy. Circulation 112:2921–2929 16275881 10.1161/CIRCULATIONAHA.105.561340

[15] Töger J, Arvidsson PM, Bock J, Kanski M, Pedrizzetti G, Carlsson M, Arheden H, Heiberg E (2018) Hemodynamic forces in the left and right ventricles of the human heart using 4D flow magnetic resonance imaging: phantom validation, reproducibility, sensitivity to respiratory gating and free analysis software. PLoS ONE 13:e0195597 29621344 10.1371/journal.pone.0195597 PMC5886587

[16] Pola K, Roijer A, Borgquist R, Ostenfeld E, Carlsson M, Bakos Z, Arheden H, Arvidsson PM (2023) Hemodynamic forces from 4D flow magnetic resonance imaging predict left ventricular remodeling following cardiac resynchronization therapy. J Cardiovasc Magn Reson 25:45 37620886 10.1186/s12968-023-00955-8 PMC10463519

[17] Itatani K, Okada T, Uejima T, Tanaka T, Ono M, Miyaji K, Takenaka K (2013) Intraventricular flow velocity vector visualization based on the continuity equation and measurements of vorticity and wall shear stress. Jpn J Appl Phys 52:07HF16

[18] Minami S, Masuda K, Stugaard M, Kamimukai T, Asanuma T, Nakatani S (2021) Noninvasive assessment of intraventricular pressure difference in left ventricular dyssynchrony using vector flow mapping. Heart Vessels 36:92–98 32632552 10.1007/s00380-020-01664-3

[19] Firstenberg MS, Smedira NG, Greenberg NL, Prior DL, McCarthy PM, Garcia MJ, Thomas JD (2001) Relationship between early diastolic intraventricular pressure gradients, an index of elastic recoil, and improvements in systolic and diastolic function. Circulation 104(Suppl 1):I330–I335 11568078 10.1161/hc37t1.094834

[20] Sørensen K, Fadnes S, Mawad W, Henry M, Flade HM, Østvik A, Myklebust TÅ, Kirkeby-Garstad I, Løvstakken L, Mertens L, Nyrnes SA (2025) Intraventricular pressure difference Estimation based on blood speckle tracking-invasive validation and early clinical application. Eur Heart J Cardiovasc Imaging 26:1346–1357 40365693 10.1093/ehjci/jeaf149 PMC12378583

[21] Uema A, Tamura Y, Uejima T, Hoshiai M, Ueno A, Nagao M, Tomoe T, Ono S, Maeno E, Mizuguchi S, Kawabe A, Sugiyama T, Yasu T (2022) Early diastolic mitral regurgitation in left ventricular aneurysm. Heart Vessels 37:683–690 34689257 10.1007/s00380-021-01958-0

[22] Warisawa T, Nour D, Seligman H, Doi S, Kuwata S, Howard JP, Rajkumar C, Cook CM, Nakayama Y, Kasahara M, Suzuki N, Matsuda H, Mizuno K, Akashi YJ (2020) Interference between pressure-wire and deployed coronary stents: insights from a bench test. Cardiovasc Revasc Med 21:765–770 31784356 10.1016/j.carrev.2019.10.026

[23] Cottens D, Ferdinande B, Polad J, Vrolix M, Ameloot K, Hendrickx I, Poels E, Maeremans J, Dens J (2022) FFR pressure wire comparative study for drift: piezo resistive versus optical sensor. Am J Cardiovasc Dis 12:42–52 35291508 PMC8918737

[24] Murayama M, Iwano H, Obokata M, Harada T, Omote K, Kagami K, Tsujinaga S, Chiba Y, Ishizaka S, Motoi K, Tamaki Y, Aoyagi H, Nakabachi M, Nishino H, Yokoyama S, Tanemura A, Okada K, Kaga S, Nishida M, Nagai T, Kurabayashi M, Anzai T (2022) Visual echocardiographic scoring system of the left ventricular filling pressure and outcomes of heart failure with preserved ejection fraction. Eur Heart J Cardiovasc Imaging 23:616–626 34694368 10.1093/ehjci/jeab208 PMC9016355

[25] Murayama M, Iwano H, Nishino H, Tsujinaga S, Nakabachi M, Yokoyama S, Aiba M, Okada K, Kaga S, Sarashina M, Chiba Y, Ishizaka S, Motoi K, Nishida M, Shibuya H, Kamiya K, Nagai T, Anzai T (2021) Simple two-dimensional echocardiographic scoring system for the Estimation of left ventricular filling pressure. J Am Soc Echocardiogr 34:723–734 33675942 10.1016/j.echo.2021.02.013

[26] Yotti R, Bermejo J, Benito Y, Antoranz JC, Desco MM, Rodríguez-Pérez D, Cortina C, Mombiela T, Barrio A, Elízaga J, Fernández-Avilés F (2011) Noninvasive Estimation of the rate of relaxation by the analysis of intraventricular pressure gradients. Circ Cardiovasc Imaging 4:94–104 21245360 10.1161/CIRCIMAGING.110.960369

[27] Tanaka T, Okada T, Nishiyama T, Seki Y (2017) Relative pressure imaging in left ventricle using ultrasonic vector flow mapping. Jpn J Appl Phys 56:07JF26

[28] Asami R, Tanaka T, Kawabata KI, Hashiba K, Okada T, Nishiyama T (2017) Accuracy and limitations of vector flow mapping: left ventricular Phantom validation using stereo particle image velocimetory. J Echocardiogr 15:57–66 27848215 10.1007/s12574-016-0321-5 PMC5429903

[29] Taniguchi N, Miyasaka Y, Suwa Y, Nakai E, Harada S, Otagaki H, Shiojima I (2024) Incremental value of diastolic wall strain in predicting heart failure events in patients with atrial fibrillation. Heart Vessels 39:785–794 38625395 10.1007/s00380-024-02401-w

[30] Gong C, Kinoshita T, Hayashida M, Hara A, Kakemizu-Watanabe M, Miyazaki S, Tabata M (2025) The role of E-wave velocity in predicting early left ventricular dysfunction and significant decline in left ventricular ejection fraction after mitral valve repair for severe chronic primary mitral regurgitation. Heart Vessels 40:320–331 39375196 10.1007/s00380-024-02468-5

[31] Peverill RE, Ngian GS, Mylrea C, Sahhar J (2021) Determinants of left ventricular structure, filling and long axis function in systemic sclerosis. PLoS ONE 16:e0258593 34679117 10.1371/journal.pone.0258593 PMC8535357

[32] Onose Y, Oki T, Tabata T, Yamada H, Ito S (1999) Assessment of the Temporal relationship between left ventricular relaxation and filling during early diastole using pulsed doppler echocardiography and tissue doppler imaging. Jpn Circ J 63:209–215 10201623 10.1253/jcj.63.209

[33] Han Y, Huang L, Li Z, Ma N, Li Q, Li Y, Wu L, Zhang X, Wu X, Che X, Zhang H (2019) Relationship between left ventricular isovolumic relaxation flow patterns and mitral inflow patterns studied by using vector flow mapping. Sci Rep 9:16264 31700142 10.1038/s41598-019-52680-x PMC6838154

[34] Uema A, Sugiyama T, Yasu T (2021) A case series of patients with early diastolic mitral regurgitation and left ventricular aneurysm. Clin Cardiol J5:1–3

[35] Park JH, Marwick TH (2011) Use and limitations of E/e’ to assess left ventricular filling pressure by echocardiography. J Cardiovasc Ultrasound 19:169–173 22259658 10.4250/jcu.2011.19.4.169 PMC3259539

[36] Nagueh SF (2020) Left ventricular diastolic function: understanding pathophysiology, diagnosis, and prognosis with echocardiography. JACC Cardiovasc Imaging 13:228–244 30982669 10.1016/j.jcmg.2018.10.038

