# Supplementary material for: Late-systolic to early-diastolic hemodynamic force analysis in ischemic heart disease using vector flow mapping and invasive pressure measurement
Source: Heart Vessels. 2026 Feb 16;41(10):641–52. doi: 10.1007/s00380-026-02664-5 (PMC13588891; doi:10.1007/s00380-026-02664-5)
Supplement: Supplementary file 1 [file 380_2026_2664_MOESM1_ESM.pdf]

Article title: Late-systolic to early-diastolic hemodynamic force analysis in ischemic heart disease using vector flow mapping and invasive pressure measurement

Journal name: Heart and Vessels

Author names: Takushi Sugiyama, Naoyuki Otani, Haruka Noma, Harunori Takahashi, Takanori Yasu, Yasuhiro Maejima

Corresponding author: Naoyuki Otani, MD, PhD
Department of Cardiology, Dokkyo Medical University Nikko Medical Center
Address: 145-1 Moritomo, Nikko city, Tochigi, 321-1298, Japan
Phone: +81-0288-23-7000
Fax: +81-0288-23-5000
E-mail: naoyuki@dokkyomed.ac.jp

**Supplementary Table S1**

**Baseline clinical characteristics of the patients in the healthy control (H) group**

| | H group (n =11) |
|---|---|
| Mean age (years) | 53 ± 5 |
| Male, n (%) | 11 (100%) |
| Hypertension, n (%) | 2 (18%) |
| Dyslipidemia, n (%) | 1 (9%) |
| Diabetes mellitus, n (%) | 2 (18%) |
| Chronic kidney disease | 0 (0%) |
| Heart failure (AHA/ACC stage C), n (%) | 0 (0%) |
| Previous cerebral infarction, n (%) | 0 (0%) |
| Peripheral artery disease, n (%) | 0 (0%) |

*ACC:* American College of Cardiology, *AHA:* American Heart Association.

**Supplementary Table S2**

**Comparison of the echocardiographic data of the patients in the healthy control (H) group and ischemic heart disease without left ventricle aneurysm group (IHD group)**

| Variables | H group (n = 11) |
|---|---|
| LVDd (mm) | 44 ± 6 |
| LVDs (mm) | 26 ± 3 |
| LVEDV (mL) | 96 ± 29 |
| LVESV (mL) | 32 ± 13 |
| EF (%) | 67 ± 5 |
| E-wave (m/sec) | 0.7 ± 0.2 |
| E/A | 0.9 ± 0.2 |
| E/e’ | 10 ± 2 |
| e’ | 7.9 ± 2.0 |
| LAVI (mL/$m^2$) | 21 ± 8 |
| LAEI (%) | 139 ± 43 |
| VMT score: n (%) | |
| 0 | 11 (100%) |
| 1 | 0 (0%) |
| 2 | 0 (0%) |
| $IVPD_{VFM}$ recoil (mmHg) | 3.0 ± 1.8 |
| $IVPD_{VFM}$ RF (mmHg) | 2.3 ± 1.0 |
| $IVPD_{VFM}$ recoil + RF (mmHg) | 5.3 ± 2.2 |

*H group:* Healthy adults without ischemic heart disease (IHD), *IHD* group: Patients with IHD without left ventricular aneurysm (LVA), *LVDd:* Left ventricular end-diastolic

diameter, *LVDs:* Left ventricular end-systolic diameter, *LVEDV:* Left ventricular end-diastolic volume, *LVESV:* Left ventricular end-systolic volume, *EF:* Ejection fraction, *E/A:* Ratio of the early (E) to late (A) ventricular filling velocities, *E/e':* Ratio of early diastolic mitral inflow velocity to early diastolic mitral annular velocity, *LAVI:* Left atrial volume index, *LAEI:* Left atrial expansion index, $IVPD_{VFM}$ *recoil*: Intraventricular pressure difference by VFM during LV recoil in end systole, $IVPD_{VFM}$ *RF*: IVPD by VFM during rapid filling, *LVEDP:* Left ventricular end-diastolic pressure, *VMT score*: Visually assessed time difference between the mitral valve and tricuspid valve opening score.

**Supplementary Figure S1. Flow diagram of patient selection for the study.**

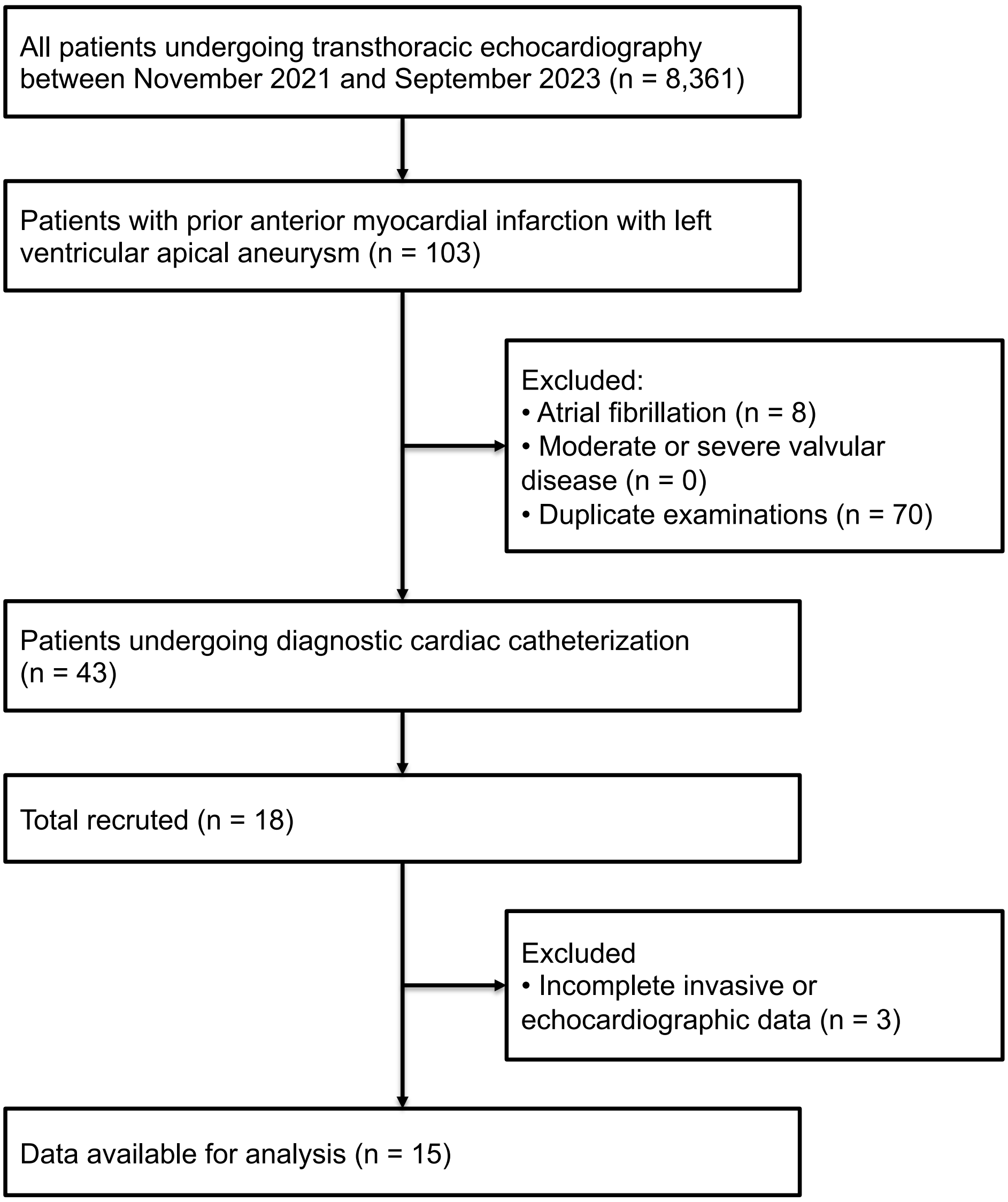


Transthoracic echocardiographic examinations performed between November 2021 and March 2024 were screened to identify patients with anterior myocardial infarction and left ventricular apical aneurysm (LVA). After applying predefined exclusion criteria and confirming the availability of cardiac catheterization data, informed consent, and complete echocardiographic and invasive measurements, 15 patients were included in the final LVA group. Patients with ischemic heart disease without apical aneurysm and healthy controls were identified during the same period as described in the Methods.

**Supplementary Figure S2. Correlation between E/A and LVEDP in patients with ischemic heart disease (IHD) without left ventricle aneurysm (LVA) (IHD group) and those with IHD with LVA (LVA group)**

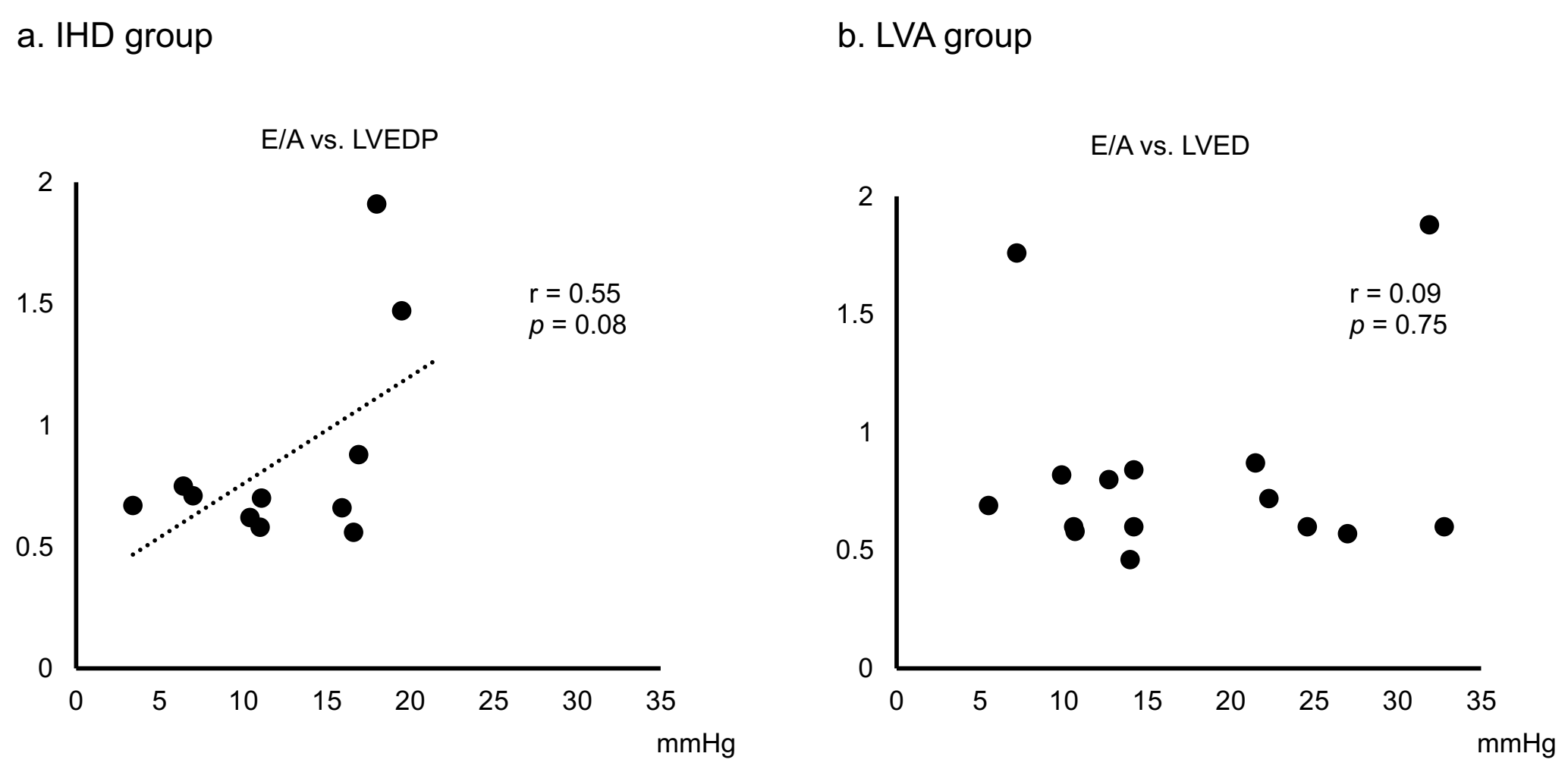


Correlation between E/A and LVEDP in IHD group (a) and LVA group (b). In IHD group, E/A showed a trend toward correlation with LVEDP (r = 0.55, P = 0.07), but no such correlation was observed in the LVA group (r = 0.02, P = 0.94).

*E/A:* Ratio of the early (E) to late (A) ventricular filling velocities, *LVEDP:* Left ventricular end-diastolic pressure.

**Supplementary Figure S3. Correlation between mitral annular e′ velocity and invasively measured LV end-diastolic pressure (LVEDP)**

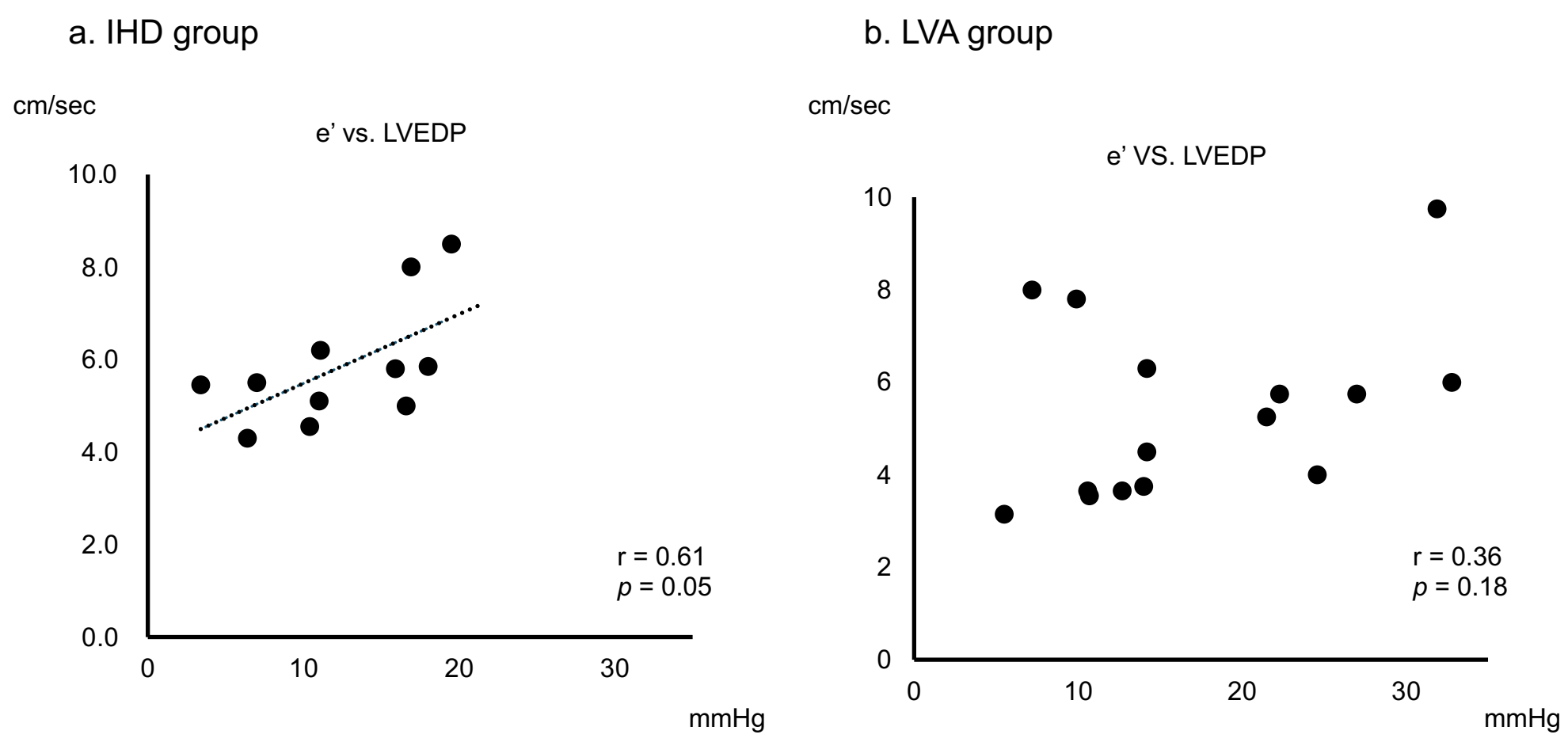


Correlation between e′ and LVEDP in the IHD group (a) and LVA group (b). In the IHD group, e′ demonstrated a modest inverse correlation with LVEDP (r = –0.61, P = 0.05). In contrast, no significant correlation was observed in the LVA group (r = –0.36, P = 0.18). Uncorrected P-values are shown.

**Supplementary Figure S4. Correlations between IVPD$_{VFM}$ parameters and echocardiographic indices**

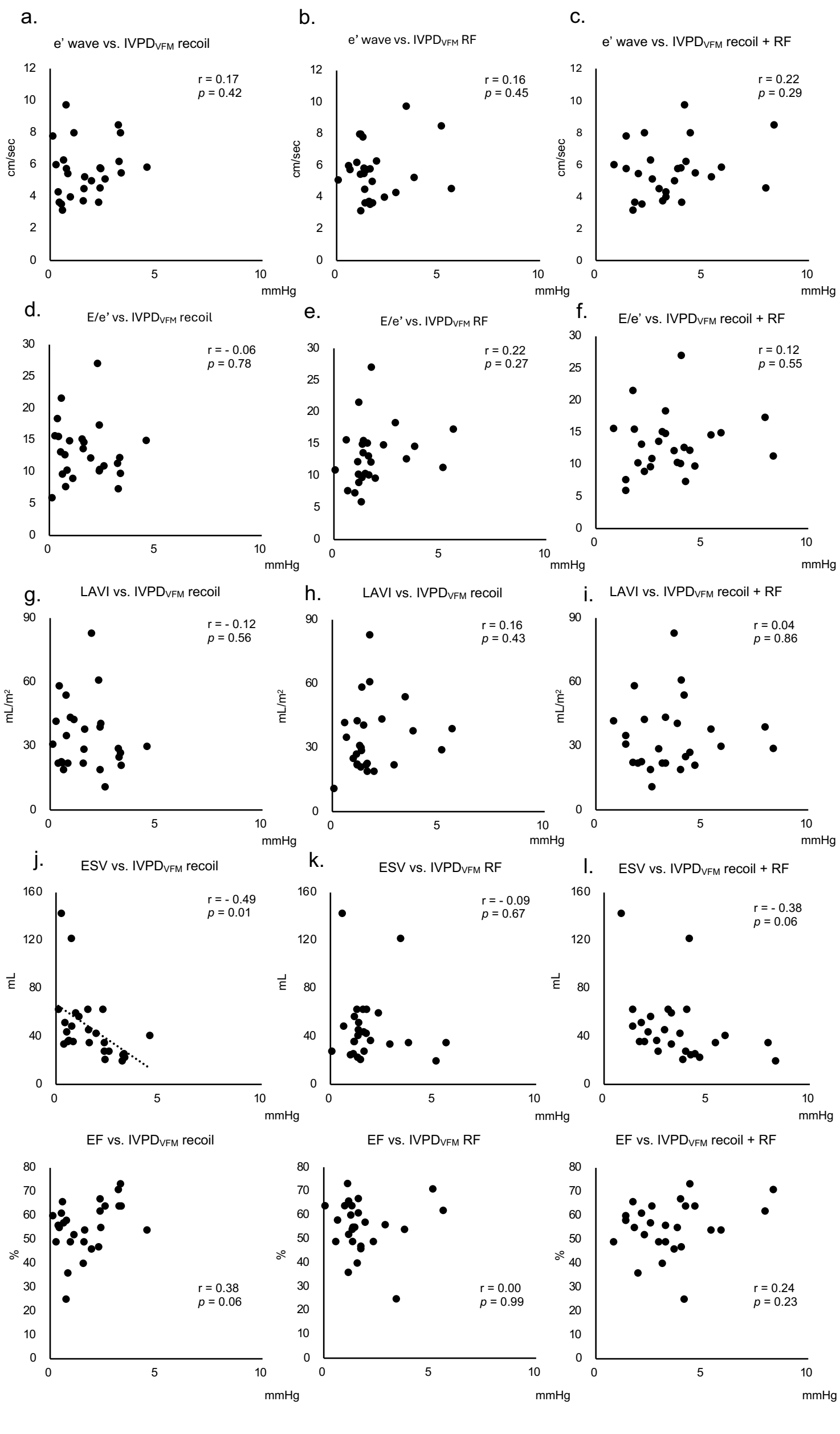


Correlation plots between IVPD$_{VFM}$ recoil, IVPD$_{VFM}$ RF, and the combined IVPD$_{VFM}$ recoil + RF and the following echocardiographic parameters: mitral annular e′ velocity, E/e′ ratio, left atrial volume index (LAVI), left ventricular end-systolic volume (LVESV), and left ventricular ejection fraction (LVEF). Among all combinations, only LVESV showed a significant inverse correlation with IVPD$_{VFM}$ recoil (r = –0.49, P = 0.01). No meaningful correlations were observed between IVPD$_{VFM}$ parameters and e′, E/e′, LAVI, or LVEF. Uncorrected P-values are shown.
